# 

**Published:** 1884-05-20

**Authors:** 


☞ Many subscribers are in arrears for subscription. Friends, *please settle at once*, and oblige R. C. WORD, Managing Editor.

---

SEE the new advertisements in this No. of McIntosh Supporter and Battery Co., A. A. Mellier, Ruckel & Co., Seven Springs Alum and Iron Mass, Ridge's Food, etc.

DEATH OF DR. WILLARD PARKER.—One of the great Surgeons of New York died in the latter part of April last. He was connected with the College of Physicians during the greater part of his professional life.

DR. S. D. GROSS, the eminent Surgeon of Philadelphia and former President of the American Medical Association, died on May 6th, 1884.

"Few men," says the New York Medical Record, "ever lived who bore greater sway over the Medical Profession than Dr. Gross has borne for many years past, and his influence was due to no art, but to the simple power of a long course of devotion to the best interest of those who delighted to do him honor."

### RECEIPTED.

1884.—Drs. Wm. Powell; J. Dilworth; W. B. Thomasson; W. N. Odium; H. Perdue; A. C. Grymes, T. J. Jones, K. H. Davis, J. V. Plummer, G. W. Luster; P. P Haines; L. N. Williams; P. T. Meigs; Robt. Lloyd; Jonathan King; A. H. Askew to June.

1883.—W. H. Johnson: W. H. Peek; J. T. Cleveland; A. N. Perkins; C. J. Church; M. M. Morton; W. T. Dowell.

1885.—E. W. Hunter; T. N. Skeen; J. S. Lee; Samuel Pool to October.

